# Precision medicine and the principle of equal treatment: a conjoint analysis

**DOI:** 10.1186/s12910-021-00625-3

**Published:** 2021-05-10

**Authors:** Eirik Joakim Tranvåg, Roger Strand, Trygve Ottersen, Ole Frithjof Norheim

**Affiliations:** 1 Bergen Centre for Ethics and Priority Setting, Department of Global Public Health and Primary Care, University of Bergen, Pb. 7804, 5020 Bergen, Norway; 2 Centre for Cancer Biomarkers, Department of Global Public Health and Primary Care, University of Bergen, 5020 Bergen, Norway; 3 Centre for the Study of the Sciences and the Humanities, University of Bergen, 5020 Bergen, Norway; 4 Oslo Group On Global Health Policy, Department of Community Medicine and Global Health and Centre for Global Health, University of Oslo, 0450 Oslo, Norway; 5 Division for Health Services, Norwegian Institute of Public Health, 0473 Oslo, Norway

**Keywords:** Priority setting, Resource allocation, Precision medicine, Cancer, Decision making

## Abstract

**Background:**

In precision medicine biomarkers stratify patients into groups that are offered different treatments, but this may conflict with the principle of equal treatment. While some patient characteristics are seen as relevant for unequal treatment and others not, it is known that they all may influence treatment decisions. How biomarkers influence these decisions is not known, nor is their ethical relevance well discussed.

**Methods:**

We distributed an email survey designed to elicit treatment preferences from Norwegian doctors working with cancer patients. In a forced-choice conjoint analysis pairs of hypothetical patients were presented, and we calculated the average marginal component effect of seven individual patient characteristics, to estimate how each of them influence doctors’ priority-setting decisions.

**Results:**

A positive biomarker status increased the probability of being allocated the new drug, while older age, severe comorbidity and reduced physical function reduced the probability. Importantly, sex, education level and smoking status had no significant influence on the decision.

**Conclusion:**

Biomarker status is perceived as relevant for priority setting decisions, alongside more well-known patient characteristics like age, physical function and comorbidity. Based on our results, we discuss a framework that can help clarify whether biomarker status should be seen as an ethically acceptable factor for providing unequal treatment to patients with the same disease.

**Supplementary Information:**

The online version contains supplementary material available at 10.1186/s12910-021-00625-3.

## Background

Stratification of patient groups into smaller subgroups or single patients is a hallmark of precision medicine, but may be perceived as discriminatory against other patients in the same group. Such practice may challenge common sense morality and the principle of formal equality, which requires equal treatment to patients that are equal in all ethically relevant aspects [1, 2]. Traditionally, potential to benefit, risk and severity of disease are considered relevant, while gender, ethnicity and religion are not. Among contested factors are patient age and personal responsibility for health [2, 3]. Despite disagreement over some of these factors’ relevance, it is known that all may influence clinical decision making and treatment allocation [4–6]. Is and should biomarker status be considered a relevant reason for unequal treatment concerning patients with the same disease?

The development of precision medicine has brought great promises [7] and by tailoring diagnostics and treatment to individual patients, the overarching motto of precision medicine can be achieved: “The right drug to the right patient at the right time” [8]. This belief is especially developed in oncology, where an increasing number of new targeted therapies are given only to a small selection of patients based on biomarkers [9]: examples are mutations that lead to upregulation of *epidermal growth factor receptor* (*EGFR*) in lung cancers [10], expression of *erb-b2 receptor tyrosine kinase 2* (*ERBB2*, also known as *HER2*) in breast cancer [11], *B-Raf proto-oncogene* (*BRAF*) mutations in melanoma [12], and expression of the *CD274 molecule* known as *Programmed death-ligand 1 (PD-L1)* expression in various cancer types [13, 14].

Biomarkers have the potential to promote better and fairer decision making [15, 16] but also lead to a range of ethical and social considerations [17]. Discrete Choice Experiments (DCEs) for cancer treatment have mostly been studied among patients [18], but studies among doctors are also prevalent [19–21]. It is of special interest to study how doctors perceive the relevance of biomarker status, as they are responsible for treatment decisions. To our knowledge, no prior study that examined treatment preferences among doctors has included biomarkers and a priority setting scenario. Accordingly, we set out to investigate Norwegian cancer doctors’ preferences when making hypothetical priority setting decisions based on individual patient characteristics. We were particularly interested in how biomarker status was perceived in relation to more traditional patient characteristics such as comorbidity and age.

## Methods

### The context

The growing costs of cancer care is a global problem [22, 23], and this is further complicated by the fact that many new cancer drugs offer only modest benefits [24]. This is also true for Norway. In 2016 the Norwegian Parliament unanimously endorsed a set of priority setting criteria for use in the health sector: health benefit and resource use (estimated as cost-effectiveness) and severity of disease (estimated as loss of quality adjusted life years (QALYs) without treatment) [25, 26]. Based on clinical trial data submitted by the drug manufacturers, a national system, the New Methods System, evaluates new drugs for reimbursement in the health care system using group level estimates of the three criteria [27]. The Norwegian Directorate of Health, together with the relevant professional associations, implement the newly approved drugs into national clinical guidelines. Clinicians must then, within these guidelines, make use of group level data combined with clinical discretion in their clinical decisions for individual patients.

Our aim was to explore this tension between group-level evidence and decisions based on individual patient characteristics by means of an email survey among Norwegian cancer doctors. In the experiment, pairs of hypothetical patients were presented to our respondents, with the information that a new cancer drug could be given to only one of the two (see supplementary material for survey questionnaire). The two patients were equal at a group level, with similar severity of disease and a cost-effectiveness of the new treatment so high that it would only just be approved for reimbursement. Their individual characteristics, like age and comorbidity, varied. We then asked the respondents, based on the information provided, to allocate one of the patients the new drug.

### Experimental design

We designed a conjoint analysis (CA), a DCE constructed to elicit stated preferences. Compared to revealed preferences, which are derived from real world observations, stated preferences are what respondents declare when asked hypothetical questions. The strengths of using stated preferences compared to revealed preferences are well-known [28]: standardised data collection makes it easier to estimate statistical relationships between patient characteristics and treatment decisions. It is easier to explore decisions involving biomarkers, as they are not yet established in clinical practice. Also, hypothetical scenarios may elicit better answers from respondents, especially concerning potentially sensitive issues like clinical priority setting. Still, we acknowledge that no hypothetical design can fully simulate real patients and clinical decisions. What respondents say that they would do may not be what they actually would do in real life decision making.

First developed for marketing research and cosumer preferences [29, 30], different types of CA have later been used in health care research [31, 32]. In this particular study, we use a modified CA developed by Hainmuller et al. [33], which has recently also been used in empirical ethics [34]. This choice-based conjoint design identifies the average marginal component effect (AMCE), the marginal effect of changing one characteristic in a patient profile averaged over the joint distribution of all the remaining patient characteristics. The AMCE can be explained with an example: if we compare a randomly drawn patient with a positive biomarker to a randomly drawn patient with a negative biomarker, how much more likely is the patient with the positive biomarker to be given the new drug?

We decided to use this modified CA because it has several strengths relevant for our survey: (1) It allows us to include a broad range of patient characteristic, in stead of pre-selecting two or three characterstics we believed would be most relevant. (2) It is able to capture multidimensional preferences, which typically are present in clinical decision making. (3) It can estimate causual effects of various patient characteristics at the same time, making more complex analysis of clinical decision making possible. (4) It does not rely on modelling assumptions and complex statistical methods.

We developed our experiment based on published recommendations [35, 36], with the modifications required by our specific type of CA. Selection of the attributes was based on both their ethical relevance for priority setting [2] and observational data from clinical practice [6] and resulted in seven patient characteristics relevant for treatment decisions in a metastatic cancer scenario. With input from clinical experts we then gave each patient characteristics a set of realistic values. Table 1 presents the included patient characteristics and levels. In our design a total of 3 × 2 × 3 × 3 × 2 × 3 × 2 = 648 different patient profiles were possible. We did not judge any of the possible combinations to be illogical, although a patient with severe comorbidity and an Eastern Cooperative Oncology Group (ECOG) performance status of 0 would perhaps have been clinically unusual (but not impossible). The experiment was tested in a pilot with eight doctors. Based on this feedback we made some minor adaptations.

**Table 1:** Patient characteristics and accompanying levels included in the conjoint analysis

Patient characteristic	Value
Patient age (years)	63
	75
	87
Biomarker status	Positive*
	Negative
ECOG performance status**	0
	1
	2
Comorbidity**	None
	Moderate
	Severe
Smoking status	Smoker
	Non-smoker
Sex	Woman
	Man
Education**	Low
	Medium
	High

*Defined as a 50% probability of better effect than average

**These characteristics were given a more detailed description. This is available in the supplementary material

The questionnaire had three sections. The first section was for the conjoint analysis. The second section asked a general question about background values shaping the priority setting for individual patients, and the third and final section asked questions about the respondents’ background. The survey is available in Additional file 1.

### Distribution and analysis

The survey was programmed and distributed, and responses were collected by Ideas2evidence, a company specialising in survey development and administration. Confirmit, a web-based survey software was used. Respondents could access the questionnaire at any time during the data collection period, using a smartphone, tablet or computer. Responses were stored in the software and exported for analysis after data collection ended.

A general invitation to participate in the survey, with an accompanying link to the questionnaire, was emailed to 1 029 potential participants in the beginning of March 2019 using email lists from three specialized medical associations and a network of gynaecological oncologists in Norway. These were selected as they represent a large majority of doctors treating cancer patients in Norway and are familiar to biomarker-based diagnostics. A reminder was emailed 2 weeks later. Data collection ended after 5 weeks.

AMCE was estimated with a linear regression model, where allocation to treatment is the dependent outcome variable and all the patient characteristics are explanatory variables. For each characteristic one level is designated as a baseline value. The statistical analysis was conducted in R/RStudio version 3.6.1 and the R-package “cregg” [37]. Dataset and syntax are available in the supplementary section (see Additional file 3 and 4).

A total of 115 participants completed our survey, giving a total of 690 observations in our sample. Characteristics of the respondents are presented in Table 2. The majority of respondents are working in the field of oncology and at university hospitals. Only 21% have less than 5 years of experience working with cancer patients, and almost half of them treat more than 20 patients in a regular week.

**Table 2:** Demographics of survey participants

*Age of participants*	
Range (years)	27–71
Mean (years)	45
*Sex*	
Female	63 (55%)
Male	52 (45%)
*Position*	
Junior	28 (24%)
Senior	84 (73%)
Other*	3 (3%)
*Specialist training*	
Oncology	78 (68%)
Pulmonology	11 (10%)
Haematology	15 (13%)
Gynaecology	9 (8%)
Other**	4 (2%)
*Working location*	
University hospital	81 (70%)
Regional hospital	17 (15%)
Local hospital	17 (15%)
*Experience working with cancer patients*	
< 5 years	24 (21%)
5–15 years	48 (42%)
> 15 years	43 (37%)
*Number of patients treated in a regular week*	
< 5	9 (8%)
5–20	50 (43%)
> 20	56 (49%)

*1 retired, 1 Ph.D. fellow, 1 junior, but in senior position

**1 pediatric oncology and haematology, 1 surgery

### Ethical approval

The study was reported to and evaluated by the Norwegian Centre for Research Data (reference number 583480). We did not collect any patient information whatsoever. Accordingly, the study was exempt of the requirement of medical ethics approval as regulated by the Norwegian Health Research Act [38]. Attached in the email invitation sent to potential participants was a link to an informed consent form and information about data protection.

## Results

**Fig. 1:**
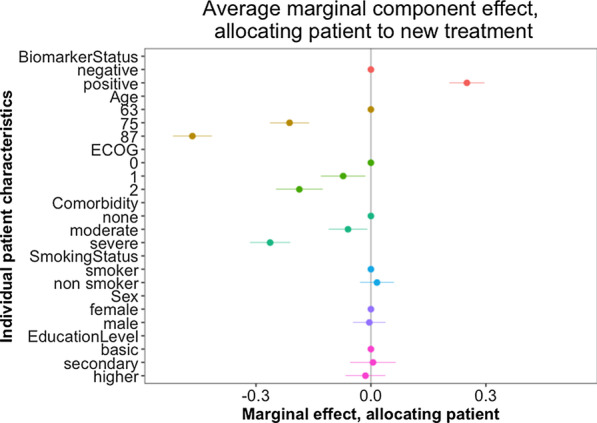
The average marginal component of changing one individual patient characteristic, compared to its baseline characteristic. Lines represent 95% confidence intervals. A positive AMCE indicates a higher probability of being allocated the new drug, while a negative AMCE indicates a lower probability

The AMCEs from our conjoint analysis are presented in Fig. 1 and show that there is a 25 percentage point (pp) increased probability that respondents would give the new drug to a patient with a positive biomarker, compared to a patient with a negative biomarker, when averaged on all other possible combinations of patient characteristics. We shall refer to these computed frequencies as “probabilities” for allocation. Biomarker status produced the third largest effect of the patient characteristics in the experiment: a patient aged 87 years has a 47 pp reduced probability of being allocated the new treatment compared to a patient aged 63, and a patient with severe comorbidity has a 26 pp reduced probability of being allocated the new treatment compared to a patient with no comorbidity. All these findings were statistically significant.

Other patient characteristics with a significant effect on the probability of being allocated the new treatment were patient age 75 years (21 pp reduced probability compared to age 63), ECOG performance status 1 and 2 (7 pp and 19 pp reduced probability compared to ECOG performance status 0) and moderate comorbidity (6 pp reduced probability compared to no comorbidity). Importantly, sex, smoking status and education level had no significant impact on the probability of being allocated the new treatment. An additional analysis of marginal means is available in Additional file 1.

**Fig. 2:**
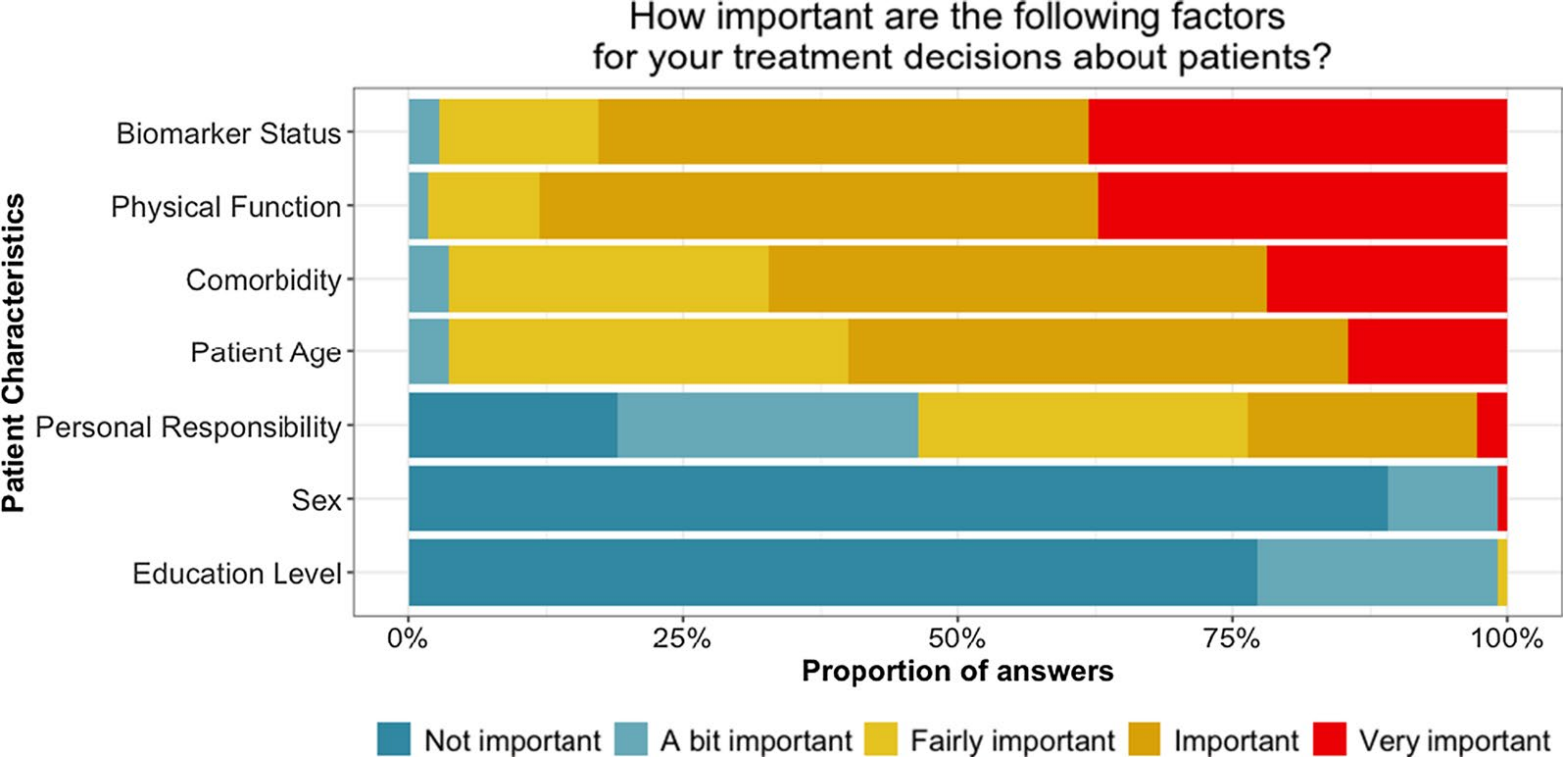
The respondents’ answers to the question “How important are the following factors for your treatment decision about patients?”

In Fig. 2 we present answers to the question about background values and show that biomarker status was rated as “important” or “very important” for treatment decisions by 83% of the respondents. None of the respondents expressed that biomarker status was unimportant. The characteristic considered as overall most important is performance status, where 88% of the respondents rated it as “important” or “very important” for their treatment decision. Comorbidity and patient age were rated as “important” or “very important” by 74% and 67%, respectively.

A large majority of the respondents considered patients’ sex (89%) and education levels (78%) as not important. The patients’ responsibility for their health was the characteristic with the most variation in ratings. 26% rated it as “important” or “very important” for the treatment decision, while 27% rated it as “a bit important” and 20% as “not important”.

## Discussion

Our main findings are that in our sample a positive biomarker status significantly increases the probability of being allocated the new cancer treatment, while older age, a higher degree of comorbidity and reduced physical function significantly reduce this probability. Sex, education and smoking status do not influence the probability. Many of these findings are in line with what is commonly seen as relevant ethical factors for treating patients unequally: A patient’s functional status and comorbidity are considered relevant because they are proxies, that could say something about the potential to benefit from treatment. Sex and education are not considered relevant. The fact that biomarker status and age are seen as relevant is particularly interesting, as if and how they should be seen as ethically relevant in decision making is disputed.

We find that a positive biomarker status increases the probability of being allocated the new treatment, compared to a negative status. Whether a biomarker can be seen as relevant depends on what the biomarker is a proxy for and how good a proxy it is. Importantly, a biomarker in itself is not inherently ethically relevant. In our survey the biomarker is a predictive marker, a proxy for health benefit. Health benefit, or the potential to benefit, has strong support in the normative literature and is also shown to be perceived as relevant by clinicians, policymakers and the public [39]. An example of a predictive biomarker is *PD-L1* in lung cancer [40]. Other biomarkers can be both predictive and prognostic, like *HER2* [11].

We find that old chronological age reduces the probability of being allocated the new treatment, compared to a younger age. This is an interesting finding, as the relevance of patient age in priority setting is heavily contested [41–44]. Many endorse the view that age can be indirectly relevant if it correlates or informs some other factor that is seen as ethically relevant (e.g. risk of disease, or survival). This view is also endorsed in the official Norwegian white paper on priority setting [45]. Accepted as indirectly relevant, chronological age is often interpreted as a proxy for biological age, which again may indicate something about the potential to benefit from a treatment [46]. However, we find that age is the single most important factor in priority setting decisions, even when comorbidity and performance status also are included. This could be interpreted as age is given an independent and direct relevance, based on normative claims like the fair innings argument.

The fair innings argument argues that at some age, originally suggested at the age of 70, one can consider oneself to have had her or his fair share of life, and that any additional time added after that age should be considered a sort of bonus [41, 47, 48]. If one dies before the age of 70 however, some injustice is being suffered because that person has not had the chance to live a reasonable length of life. The fair innings argument then requires efforts and priorities to be made to give as many as possible the chance to live until that age, while those who has passed that age, should be given less priority. Such reasoning is much more controversial [49] that the indirect use of age in priority setting and may be seen as ageism [50]. How our respondents reasoned about age in their priority setting decisions are difficult to assess. In our results there seem to be discrepancy between the important role given to age in our experiment and how important the respondents have replied when directly asked about the importance of age. One possible answer to this may that the hypothetical scenarios elicit other and perhaps more true answers from respondents on controversial issues like age and priority setting [28].

A common view is that treating equal patients unequally based on their age can be ethically acceptable if patient age indirectly is a proxy for other ethically relevant factors (such as treatment benefit), and if this correlation is strong. We argue that a similar approach is also true for biomarkers: treating equal patients unequally based on a predictive biomarker is only acceptable if the biomarker predicts treatment effect, and, importantly, if this prediction is of good quality. A similar argument would also be true for prognostic biomarkers. Such concerns may explain why respondents in our survey attach only moderate importance to biomarker status, as they may have felt uncertain about the quality of the generic biomarker presented in the survey. Further work could investigate how doctors process and use information about biomarker status and patient age in their clinical reasoning.

Ian Majewski and René Bernards have proposed three key aspects of biomarker tests when considering its regulatory trajectory [51], and these are also useful for judging a biomarker’s quality in clinical decision making [16]. A biomarker test should have analytical validity, meaning that it must be reproducible, accurate and validated, so that it measures what it is supposed to measure and the measurements are consistent. If a biomarker test demonstrates significant variations in its analytical validity, its claim to be an ethically relevant factor for decision making would weaken.

A good biomarker should also be clinically valid, meaning that it must provide clinically useful information relevant for the decision at hand. A prediction of treatment response is clearly relevant. In our survey, we informed the respondents that a positive biomarker status gave “a 50% probability of an effect better than the average”. This may be seen as too general a prediction, but this was deliberate. The survey was distributed to doctors with different specialties treating different types of cancer, so we wanted a generic experiment. The breadth in available biomarkers is large, and the average benefit from many new cancer drugs is modest [52, 53]. The current discussion about surrogate endpoints in precision oncology [54] is also important for a biomarker’s clinical validity. If a biomarker predicts tumour shrinkage or a pathological complete response, it is not clear how this should inform a treatment decision without evidence of these surrogate markers also influencing survival [55] or quality of life [56].

A biomarker should also have clinical utility, that is, actually influence decision making. This depends both on the biomarker itself and the context in which it is used. A test that has good analytical and clinical validity, as discussed above, may inform clinical decisions in a legitimate and ethical way, but this is also context dependent. Are tests and drugs available to all patients? Does the health care system have guidelines or formal processes that guide biomarker use in clinical decisions? How does tradition and organizational culture influence decisions? And do decision makers accept and trust biomarkers as part of their input into clinical decisions? This latter question is one that we explore in this article.

We believe our findings have three important implications. First, the results from our hypothetical stated choice experiment show that in our sample doctors seem to accept biomarker status as relevant for treatment decisions, but it is not seen as the most important characteristic. This may illustrate valid concers about uncertainty. Precision medicine may have led to more precise diagnostics and targeted treatments, but paradoxically, the evidence supporting it is, at present, often less precise [57]. This uncertainty about evidence in precision oncology also translates into clinical decision making [58].

Second, doctors have to balance the competing concerns for equality with the best available treatment for individual patients, all with an increasing degree of uncertainty. How they reason and make decisions needs more research and attention as their ability to navigate in this ethically and clinically challenging landscape is of great importance: For patients with advanced cancer, access to potentially beneficial treatment can be a matter of life and death. In health care systems, the principle of equality is a matter of justice, solidarity and legitimacy [59, 60].

Third, we wish to encourage a broader debate about individual biomarkers’ validity and utility. It is well-known that e.g. *PD-L1* as a predictive biomarker has significant shortcommings in its analytical and clinical validity [61, 62], but it is still part of clinical practice and guidelines [63]. Seemingly technical decisions on tissue fixation, assay properties and thresholds for positive tests also raise important normative challenges [17]. Biomarkers should therefore not be automatically integrated and accepted in clinical decision making. A highly sophisticated biomarker will not automatically improve health, nor promote fair priority setting [64].

Using Majewski and Bernard’s framework to evaluate individual biomarkers could be a step forward to guide a fair implementation of biomarkers and precision oncology: if a biomarker is analytically and clinically valid, and provides clinical utility; it can be seen as an ethically relevant factor for providing treatment to some patients while denying it to others, even if they have the same disease. And importantly, to base treatment decisions on a flawed and poor quality biomarker should be seen as unethical and in conflict with the principle of equal treatment.

### Strengths and limitations of the study

Given the momentum and strong attention to precision medicine, we believe our findings are timely and important. The relevance of biomarker status and age in priority setting are disputed but seem to be perceived as relevant in our sample. To our knowledge, this is the first survey to include biomarker status in a priority setting scenario with expensive cancer drugs.

Conjoint analysis is a well-known method from other fields and has several advantages. A potential limitation for this method is its reliance on a random and uniform distribution of patient profiles used in the conjoint analysis [65]. The median age of incidence for all cancers in Norway is 69 years, making patients aged 87 less frequent than patients aged 75 and 63. However, although not uniformly distributed in real clinical practice, all our characteristics and levels were within recommendations in clinical cancer guidelines in Norway, making all possible combinations realistic.

As this experiment was conducted in Norway, our results might not be transferable into other contexts with other frameworks for priority settings. However, limited clinical benefit and high costs of cancer medicine is a global challenge [66, 67], which makes the circumstances in our experiment fairly applicable to many other settings. We acknowledge the importance of shared decision making and that an informed and autonomous patient should be part of the final treatment decision. In our hypothetical experiment we decided to exclude this as a factor in order to isolate and explore the opinions of the doctors.

Our estimated response rate of 11% may invite questions about the validity of our results, challenges well-known for email surveys [68]. Low response rates in surveys among doctors is a recognized problem [69], but it is also argued that responding and non-responding doctors share many similar characteristics [70]. Nevertheless, the results from our sample should not be generalized. Our convenience sampling strategy, low response rate and missing information about non-respondents limit the external validity of our findings. Therefore this should be seen as a first exploratory study to map the use of biomarker status alongside other patient characteristics in priority setting decisions. We believe our results can serve as a useful base for discussion and generate hypotheses for further research.

## Conclusion

In our sample biomarker status is perceived as relevant for priority setting decisions, alongside other more well-known patient characteristics like age, physical function and comorbidity. Whether biomarkers should be used as a factor for stratifying patients and providing unequal treatment should depend on the proprerties of the biomarker: Biomarkers with sufficient analytical and clinical validity and clinical utility may be seen as an ethically relevant factor for giving unequal treatment to patients with the same disease.

## Supplementary Information

**Additional file 1**. An English translation of the full survey.

**Additional file 2**. An analysis of marginal means as a supplementary to the average marginal component effect presented in the results section.

**Additional file 3**. The syntax used for analysing the dataset, used in the software R.

**Additional file 4**. The data collected in the suryvey, except personal data about participants that cannot be shared. The file can be opened in R by using the syntax in Additional file 3.

## Data Availability

The dataset supporting the conclusions of this article is included within the article and its additional files. An except is demographic data collected from respondents on age, workplace etc. This cannot be shared due to privacy law. The R syntax used to analyze the data is also available in the supplementary materials.

## Abbreviations

AMCE: Average marginal component effect
BRAF: B-Raf proto oncogene
CA: Conjoint analysis
CD274: Cluster of differentiation 274
DCE: Discrete choice experiment
ECOG: Eastern Cooperative Oncology Group
EGFR: Epidermal growth factor receptor
ERBB2: Erb-b2 receptor tyrosine kinase 2
HER2: Human epidermal growth factor receptor 2
PD-L1: Programmed death-ligand 1
PP: Percentage points
QALY: Quality adjusted life years
