# Supplementary material for: Precision medicine and the principle of equal treatment: a conjoint analysis
Source: BMC Med Ethics. 2021 May 10;22:55. doi: 10.1186/s12910-021-00625-3 (PMC8108369; doi:10.1186/s12910-021-00625-3)
Supplement: Supplementary file 1 — Additional file 1. An English translation of the full survey. [file 12910_2021_625_MOESM1_ESM.docx]

Article title:
Precision Medicine and the Principle of Equal Treatment: a Conjoint Analysis

Authors:

Eirik Joakim Tranvåg, Roger Strand, Trygve Ottersen, Ole Frithjof Norheim Norheim

Supplementary file:

English translation of survey

Page 1

Welcome to this survey. Estimated time use is 15 minutes

The survey has the following structure:

1. Introduction and explanations
2. Six tasks where you must prioritize between two patients
3. Two questions about the reasons for your choices
4. Six questions about demography
5. An open field for comments and questions

The survey is part of Eirik Joakim Tranvåg’s PhD project named “The new era of personalized cancer diagnostics and therapy: seeking new roles for age and biomarkers in clinical decision making.” The project is a collaboration between the Institute of Global Public Health and Primary Care and the Centre for Cancer Biomarkers, both at the University of Bergen.

Page 2

You will be given information about pairs of patients. The introduction and framing around the patients are the same in all tasks. The only variables are in the table which provides information about individual patient characteristics.

Imagine that you with the information available must make a choice between two patients where only one can be treated with a new drug. The new drug is approved and available for all patients independent of biomarker status, but there is studies available that demonstrate that biomarker positive patients has a 50 % increased probability of achieving better effect that average.

Biomarkers will be a central part of personalized medicine and can in the coming years play a more important role at an individual level. We want to investigate how you reason in such decision making situations.

Page 3

Some expressions and definitions used in this survey: the following definitions are available in the survey by directing the pointer at the word, or by clicking it if you use a smartphone or a tablet.

Biomarker result:

The answer to an individual patient’s biomarker test.

A positive result indicates that the patient with a 50 % probability will achieve better effect than average. A negative result indicates that the patient will have an average effect.

Performance status (ECOG)

The individual patient’s physical function, estimated using the ECOG-scale from 0 - 5.

0 - able to perform any normal day activities, without limitation

1 - not able to perform physically demanding activities, but ambulatory and able to perform light work

2 - ambulatory and able to all self care, but unable to carry out any work activities. Up and about for > 50 % of waking hours

Comorbidity

Healthy - no previous disease

Moderate - diabetes mellitus type 2 and hypertension treated with antihypertensive

Severe - history with a transient ischemic attack and a coronary PCI, and hip osteoarthritis

Page 4 - 9

Imagine that your department orders staff to more rationing of new and expensive cancer drugs to patients.

Give this, you must choose between two patients. One patient will receive the new drug, while the other will receive what has been seen as standard treatment.

Both patients have the same condition, and at a group level, expected average remaining life expectancy with standard treatment is one year with good quality of life. The new drug costs 400 000 NOK (around 33 000 GBP / 37 000 EUR) and provides on average six extra months of life. In addition there is the following information available:

|  | **Patient A** | **Patient B** |
| --- | --- | --- |
| Biomarker status | Negative | Positive |
| ECOG performance score | 2 | 0 |
| Patient age | 63 years | 87 years |
| Sex | Female | Male |
| Comorbidity | Healthy | Severe |
| Smoking status | Smoker | Non-smoker |
| Education level | Low | High |

Given the information available, to whom would you allocate the new drug? [Choose A or B]

*

Comment: Each responder will be presented six pages with the same introduction and table, but where the variables inside the table will be randomly assigned from these values:

Biomarker status: positive or negative

ECOG performance score: 0, 1, 2

Patient age: 63 years, 75 years, 87 years

Sex: female, male

Comorbidity: health, moderate, severe

Smoking status: smoker, non-smoker

Education level: primary school, secondary school, college or university

Page 10

Follw-up questions:

Information about health benefit, resource use and severity of disease are available as average data on a group level.

How important are the following factors for your decision making for individual patients?

|  | **Not important** | **Somewhat important** | **Fairly important** | **Important** | **Very important** |
| --- | --- | --- | --- | --- | --- |
| *Patient age* |  |  |  |  |  |
| *Biomarker status* |  |  |  |  |  |
| *Patient’s function* |  |  |  |  |  |
| *Sex* |  |  |  |  |  |
| *Comorbidity* |  |  |  |  |  |
| *Personal responsibility* |  |  |  |  |  |
| *Education level* |  |  |  |  |  |

From what age do you consider a patient to be old? - [select age]

Page 11

Questions about you:

How old are you? - [type age]

What sex are you? - [choose female or male]

What position are you in? - [choose junior doctor, consultant or other]

Which area do you work in? - [oncology, pulmonology, hematology, internal medicine, gynecology, other]

Where do you work? - [university hospital, regional hospital, local hospital, other]

How many years of experience do you have treating cancer patients? - [<5, 5-15, >15]

How many cancer patients do you treat during a normal week at work? - [less than 5, 5-20, more than 20]

Do you have any questions or comments?
