# Supplementary material for: Precision medicine and the principle of equal treatment: a conjoint analysis
Source: BMC Med Ethics. 2021 May 10;22:55. doi: 10.1186/s12910-021-00625-3 (PMC8108369; doi:10.1186/s12910-021-00625-3)
Supplement: Supplementary file 2 — Additional file 2. An analysis of marginal means as a supplementary to the average marginal component effect presented in the results section. [file 12910_2021_625_MOESM2_ESM.docx]

Article title:
Precision Medicine and the Principle of Equal Treatment: a Conjoint Analysis

Authors:

Eirik Joakim Tranvåg, Roger Strand, Trygve Ottersen, Ole Frithjof Norheim Norheim

Supplementary file:

Appendix A: Analysis of Marginal Means

Marginal mean (MM) is an analysis that estimates each individual patient factor’s effect on the probability of being allocated to the new drug, which provides useful supplementary information about the responses. While AMCE values estimates a relative difference related to a reference characteristic (like the effect of a patient being 87 years old, compared to being 63), MM estimate the absolute effect of each characteristic.


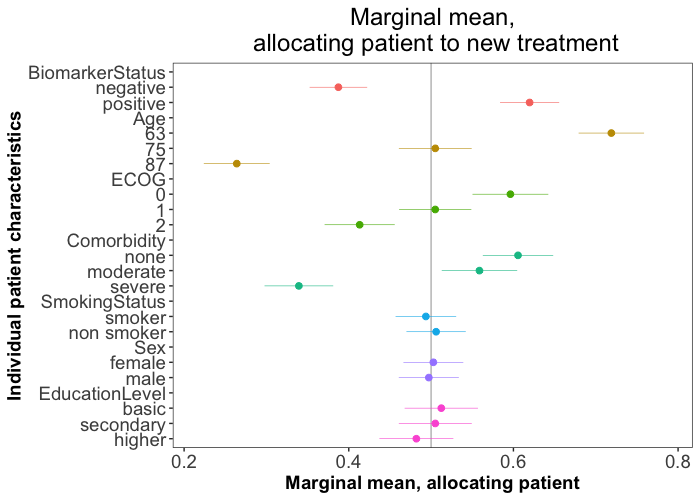


**Figure Appendix1**. Marginal means for all individual patient characteristics, describing the probability for responders to allocate the new drug to patients. A marginal mean of 0.5 indicates no effect on the allocation. A marginal mean above 0.5 indicates increased probability, while marginal means below 0.5 indicates reduced probabilities.

In Figure Appendix1 we present the marginal means for each individual patient characteristic and it shows that a positive biomarker test significantly increases the probability of being allocated to the new drug, while a negative test reduces the probability. Other characteristics that significantly increase the probability of allocation are age 63 years, ECOG performance status 0, and none and mild degrees of comorbidity. Age 87 years, ECOG performance status 2 and severe comorbidity reduce the probability of allocation. Sex, education and smoking status, and also age 75 years and ECOG performance status 1, have no significant effect on the probability of being allocated to the new treatment.
